# The Limit of State Control of Medical Practice

**Published:** 1909-09

**Authors:** 


**A Monthly Review of Medicine and Surgery.**

*EDITOR*

WILLIAM WARREN POTTER, M. D.

All communications, whether of a literary or business nature, books for review and exchanges, should be addressed to the editor, 238 DELAWARE AVENUE, BUFFALO, N.Y.

VOL. LXV. SEPTEMBER, 1909. No. 2

### The Limit of State Control of Medical Practice.

IT must appear evident to all who have paid careful attention to the question, that the limit of the functions of the state has been reached, at least nearly if not quite, in its control of medical practice. The original intent of the law regulating the practice of medicine in New York was to protect the people against unqualified men who were able, through one means or another, to obtain medical diplomas, the diploma then being the license to practise.

The statute was framed with a view to accomplish the purpose without being onerous to candidates for state license. It was believed that the methods established were sufficient to create adequate standards of preliminary education, of medical training, and of state examination to remedy heretofore existing evils. The experiences growing out of fifteen or eighteen years' administration of the law would seem to justify the further belief that it would be unwise at this time to erect additional barriers to entry into medical practice; in short, that to increase the statutory requirements for beginning the practice of medicine in this state would neither be in keeping with the best interests of the candidates, nor those of the state.

We are aware that many persons of experience advocate the addition of laboratory, clinical, and oral examinations to the methods already in vogue. This is all very well in theory, but we regard it as misleading to call them "practical" additions to present examinations. Certainly in the State of New York with 600 or 700 candidates every year, it would be most impractical to establish such additional requirements. For example, at the June examination, with nearly or quite 350 candidates for state license to deal with, how could any clinic be established for such a number without entailing an expense that would be unnecessary if not impossible to meet? It is quite out of the question to expect the state to appropriate money for the purpose, and to tax the candidate with additional fees would be unjust.

We cannot resist the conviction that these so-called practical examinations, are advocated through a mistaken idea of the real purposes of the state in exercising control of medical education. These persons in general are teachers of more or less ability as such, but in our view fail to realise the fact that the limitations of control already have been reached in many states, while in others the standards are advancing well toward the limit that can be maintained, without overstraining the tension existing between state and candidate.

The *Medical Review of Reviews* recently discussed this question in its editorial columns with such rare knowledge of the whole subject that we take pleasure in reproducing its remarks.

STATE MEDICAL EXAMINATIONS.

When the proposition is seriously made to require both an oral and written examination and also bedside demonstrations of all applicants for license to practise, it is time for us to reconsider the purpose for which these state examinations were instituted. They were primarily undertaken as a check upon medical colleges, some of whom, it was well known, granted diplomas to illterate and in many cases grossly incompetent men. Doctors came to the United States with diplomas which they had bought from a junk man or taken from the office of the deceased owner, and some check was needed upon such infamous proceedings.

The present examination in vogue in New York State has entirely overcome these troubles. Every foreign applicant must pass his examination in the English language. Our own medical schools have so improved their methods of instruction and terms of study that today a medical diploma is a guarantee of ability to practise medicine.

It does not appear to us necessary to greatly increase the burden placed upon applicants for admission to practise medicine.

Uniformity of requirements is rapidly being secured in the different states and will be accomplished without Federal interference, and all interested should be content when that goal is reached without agitating the question of new and more drastic methods of examination.

It should be remembered that the state through its control safeguards the entrance to the study of medicine, bidding the incompetent, the uneducated to turn aside, which is all-important even more so than other features of its supervision. Again, the state does not usurp the function of the college, but leaves to the latter the duty of instructing, of teaching, leaves these to the medical schools exclusively, absolutely. It is in the schools that clinical and laboratory instruction is given—must be given. The student pays for it and it is not just to make him pay for it the second time.

When all interested become familiar with the fact that the state merely exercises a supervisory office, the object of which is to determine if the medical school is doing its whole duty toward the pupil, the state examination will be better understood and more kindly accepted as a benefit to all concerned—pupil, school, and state board.

---

THIS is what is being done in New York to prevent flies from spreading infection. A notice is published in the papers and by circulars giving information and warning as follows:

Flies are among the most dangerous insects we have. They walk over filthy places like sewers and garbage cans, and after eating the filthy food which they find there, they come into your house and walk on the food you eat, carrying on their feet the germs which live in filth. These germs are not only filthy and disgusting, but many of them cause such diseases as tuberculosis, typhoid fever, cholera infantum and summer complaint. When the flies bring them from some dirty place to your food, or leave some of them when they crawl on your face or hands, you may swallow these germs without knowing it and be taken ill with one of these diseases.

Screens should be placed at doors and windows during the warm weather, to keep the flies out of the house. If you cannot screen all the rooms, you should screen those in which food is kept, and if any one is sick in the house, flies should be kept from the sickroom.

No one should buy or use meats, fruits, candy or other food which is left in front of stores or anywhere else where flies may feed and walk upon it or where the dust of the street may settle upon it.

Flies lay their eggs chiefly in stable manure, and if this is left without screens or other covers to keep the flies away, great numbers will be hatched in every stable. If you know of stores where food is not covered from flies, of stables that have swarms of them around, write to the Board of Health about them, and the board will make the storekeepers or stablemen obey its rules. But before you report other people for being careless and dirty and so making it possible for flies to become a nuisance, be sure that your own house is clean, and that no garbage cans or boxes are left uncovered to attract flies.

If you and all the people you know will follow this advice, you will be doing your part in the national movement to exterminate the house-fly and thereby remove one of the greatest dangers to health.

Rules for dealing with the fly nuisance may be had on application to The Merchants' Association's committee on Pollution of the Waters of New York, 66-72 Lafayette Street. Edward Hatch, Jr., Chairman; J. Pierpont Morgan, John Y. Cuyler, C. E. A. Vander Veer, M.D., Daniel D. Jackson.

The worst of the fly season is yet to come. Let Buffalo take prompt and strenuous action.

---

BECAUSE of the part he played in connection with the international exposition in Bordeaux, France, in 1907, Dr. Harvey W. Wiley, chief of the Bureau of Chemistry, Department of Agriculture, has had conferred upon him by the President of France the cross of the Legion of Honor. Dr. Wiley was officially informed of this action through the French Embassy at Washington, August 4, 1909. It will be necessary for Dr. Wiley to obtain authority from Congress to accept the decoration, which will be asked through the Secretary of Agriculture.

---

SUGAR instead of alcohol as a rapid reliever of fatigue is something which we are only just beginning to appreciate, and which goes surprisingly far already, according to *Success Magazine.* It has been incorporated into the most hard-headed, cold-blooded, matter-of-fact diet on earth, the German army rations, especially the forced-march emergency ration. No other food of its bulk can take its place. It is the belief of careful observers of men, particularly in the tropics, that the larger the amount of sugar, and sugar-containing foods they are supplied with, the less alcohol and other stimulants they will crave. For instance the United States Government now buys the best and purest of candy by the ton, and ships it to the Philippines, to be supplied to the canteens and messes, finding that its use diminishes the craving for native brandy; and it has long been a matter of comment from thoughtful observers that the amount of drunkenness of a race or class is in inverse ratio to the amount of sugar it consumes.

---

GOVERNOR FORT, of New Jersey, in an address to a large number of physicians, whom he had invited to meet Major General Leonard Wood at luncheon at the state camp at Sea Girt, August 5, 1909, condemned in caustic terms the use of the so-called medical expert as a witness. He said:

During my term in the Supreme Court of New Jersey it was my privilege to see much of the medical men of the state. Experience has given me great faith in the medical witness—not, however, the medical expert witness, so-called. My faith in this latter class has never been great, and observation has only tended to lessen my opinion of him. Courts and juries give great weight to the testimony of the local medical practitioner, but very little to the man who appears as an expert witness, especially in homicide cases.

The man who testifies today in a homicide case that the defendant is insane, and shortly afterward testifies that he is sane, is neither influential with the courts nor respected by them. He is an injury to the profession and to the cause of justice. The sooner the doctors frown down all such men, the better it will be for the profession. The state should aid you in this all it can.

The Governor's remarks created a mild sensation, since Dr. Britton D. Evans, one of the experts in the Thaw case, is a prominent New Jersey alienist, being head of the state insane asylum at Morris Plains. Among the Governor's listeners were Dr. Mallon and Fisher, two of Dr. Evan's assistants at Morris Plains.

---

MISS MARY HARRIMAN, eldest daughter of E. H. Harriman, has taken one of her father's Erie ferryboats and turned it into a vessel to fight tuberculosis. She has presented the boat to the Brooklyn Committee on the Prevention of Tuberculosis and the Brooklyn Red Cross Society. It went into commission as a part of the Red Cross navy on July 1, since which time its flag has flown over an anchorage off Columbia Street, Brooklyn.

Hammocks, steamer chairs and other conveniences for out-in-the-air sleeping are arranged for the accommodation of 300 men, women and children. Three meals a day are served on the boat and between meals the patients get all the milk and eggs they are able to eat.

For the commissary department, Miss Harriman forages on her father's country place at Arden, where the milk is famous and the farm products the best money can command. A free bus is run to the boat from Brooklyn stations for those who cannot pay car fare.

The boat is so anchored as to command the full benefit of the bay breezes, with a fine view of the entire waterscape in which the statue of Liberty is the center figure. Attendants and physicians are provided, and every necessity of a floating hospital is met.

